# Update for diagnosis and treatment of syndrome after cardiac injury: a mini-review

**DOI:** 10.3389/fcvm.2025.1526671

**Published:** 2025-02-05

**Authors:** Ruihui Lai, Tan Xu

**Affiliations:** ^1^ Department of Cardiology, Peking University Shenzhen Hospital, Shenzhen, China; ^2^ Department of Cardiology, Shantou University Medical College, Shantou, China

**Keywords:** post-cardiac injury syndrome, pericarditis, autoimmune-mediated, cardiac implantable electronic device placement, percutaneous coronary intervention

## Abstract

Post-Cardiac Injury Syndrome (PCIS) refers to a collective term encompassing post-myocardial infarction syndrome, post-pericardiotomy syndrome, and post-traumatic pericarditis. With an aging population, the incidence of PCIS is on the rise annually. This condition is considered to be an autoimmune-mediated inflammatory response leading to pericarditis as the primary manifestation of the cardiac disease. Early diagnosis and effective treatment can improve the quality of life and prognosis for PCIS patients. This review aims to provide a comprehensive explicit of the epidemiological characteristics, relevant pathophysiological mechanisms, diagnostic methods, and treatment strategies associated with PCIS, further fostering a deeper understanding and promoting advancements in the prevention and treatment of PCIS.

## Graphical Abstract

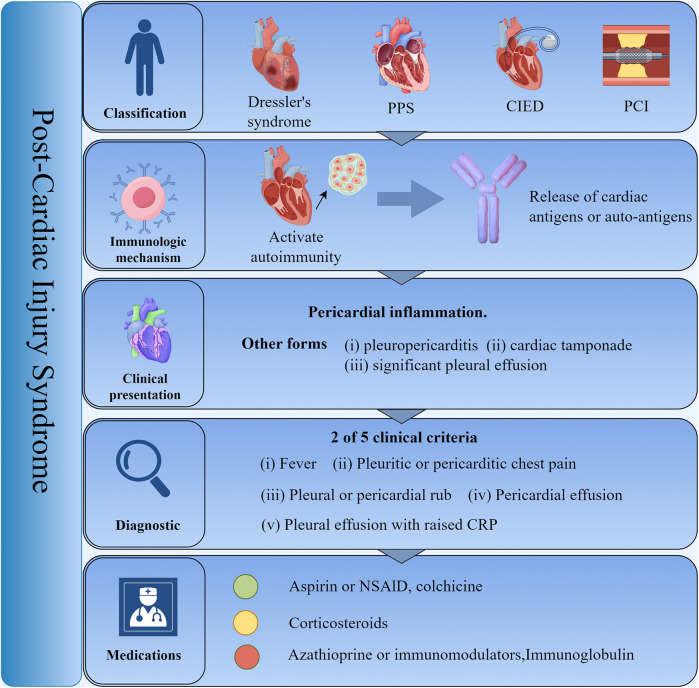

Post-Cardiac injury syndrome. PCIS refers to a collective term including 4 types, Dressler's syndrome, PPS, and post-traumatic pericarditis. This condition is considered to be an autoimmune-mediated inflammatory response leading to pericarditis as the primary manifestation of the cardiac disease. The first-line treatment options for PCIS are nonsteroidal anti-inflammatory drugs (NSAIDs) and colchicine. PICS, post-cardiac injury syndrome; PPS, post-pericardiotomy syndrome; CIED, cardiac implantable electronic device placement; NSAIDs, nonsteroidal anti-inflammatory drugs; PCI, percutaneous coronary intervention; CRP, C-reactive protein.

## Introduction

Post-cardiac injury syndrome (PCIS) refers to a condition that occurs after the heart suffering some form of damage, affecting the pericardium, epicardium, and myocardium. Clinical manifestations may include chest pain, pericardial effusion, arrhythmias, fatigue, and dyspnea (1). By definition, PCIS includes post-myocardial infarction syndrome (also known as Dressler's syndrome); post-pericardiotomy syndrome (PPS); and post-traumatic pericarditis caused by iatrogenic procedures such as percutaneous coronary intervention (PCI), cardiac implantable electronic device placement (CIED), and ablation surgery, as well as accidental injuries (e.g., stab wounds, chest compression impacts). If not treated promptly, it can lead to pericardial constriction or tamponade, resulting in increased hospitalization rates and medical costs, while also reducing the quality of life for patients.

## Epidemiological study

PCIS has become a global health concern. Before reperfusion treatment, the incidence of Dressler syndrome, one type of PCIS, was estimated at 3%–5% (2). With the introduction of primary PCI and emergency coronary artery bypass surgery, combined with early reperfusion and standardized medical treatments, the size of myocardial infarction in patients has generally decreased, further reducing this ratio to less than 1%. This indicates that with standardized quality, cardiac injury in patient's post-myocardial infarction has greatly improved.

The incidence of pericarditis caused by iatrogenic trauma depends on the intervention factors. Some research indicates that a significant number of patients develop PCIS after cardiac surgery; in a study of patients undergoing non-emergency valve surgery, out of 822 patients, 119 (14.5%) developed PPS (3). A multicenter study on patients undergoing pericardial drainage surgery showed that out of 124 patients followed, 21 (16. 9%) developed PCIS; after implantation of cardiac devices, it's about 1%–2% (4); the risk after coronary intervention is only about 0.2% (5), and procedures such as radiofrequency ablation and Swan-Ganz catheter insertion can also induce varying degrees of myocardial injury. Compared to adults, children, particularly those with congenital heart diseases, have a relatively high incidence of PCIS after undergoing cardiac surgery. Some studies indicate that the incidence of PCIS in children ranges from 10% to 28% (6).

Additionally, the incidence of PCIS may vary between different countries and regions. Developed countries generally have a higher incidence of PCIS due to aging populations, lifestyle changes, and the expansion of indications for percutaneous cardiac interventions and surgeries, while it is relatively lower in developing countries. However, as the economies of developing countries grow rapidly and lifestyles change, the incidence of PCIS is also expected to increase annually.

## Pathophysiology

The underlying mechanisms of PCIS have not yet been fully elucidated, and its pathophysiology continues to evolve. The widely accepted perspective is that myocardial injury releases myocardial antigens, which interact with myocardial antibodies to form immune complexes, thereby inducing a series of inflammatory responses (7). The autoimmune nature of PCIS is pinpointed by clinical characteristics. For instance, there is a latency period between injury and symptoms, and inflammatory markers are elevated. Additionally, PCIS responds well to non-steroidal anti-inflammatory drugs (NSAIDs) and tends to recur (8).

Early injury triggers the release of cardiac antigens, which then activates the immune response. The immune complexes formed subsequently deposit in the pericardium, pleura, and lungs, thereby inducing an inflammatory response and ultimately leading to a highly inflammatory state, increasing the risk of some individuals developing PCIS (9). Studies have found that the ratios of preoperative and postoperative anti-actin antibodies and anti-myoglobin antibodies are significantly correlated with the incidence of post-myocardial injury syndrome (10). However, the exact role of these autoantibodies in pericardial and vascular injury has not been fully elucidated and remains controversial, possibly representing only an incidental finding (11).

Moreover, PCIS involves multiple complex and interconnected processes, including viral infections and non-autoimmune reactions, which are being extensively explored (12). Studies on orthotopic heart transplantations have highlighted PCIS in immunosuppressed children, leading some researchers to propose that the syndrome may not originate from an autoimmune response. PCIS have shown seasonal variations, with increased viral titers and anti-cardiac antibody levels suggesting a potential causal relationship with viral infections. A deeper understanding of these mechanisms will aid in developing more effective prevention and treatment strategies to address the challenges posed by cardiac injury syndrome.

## Clinical presentation

Patients with PCIS exhibit symptoms and signs similar to those with pericarditis, primarily presenting with fever, chest pain, elevated inflammatory markers, pericardial effusion, and pleural effusion.

The primary characteristic involves damage or invasive procedures to the pericardium and myocardium. There is a latency period from injury to the manifestation of pericarditis or pericardial effusion, which varies individually and typically lasts from several weeks to months (13). In a study involving 360 patients with cardiac surgery, 15% developed PCIS. Of these cases, 79.6% occurred within the first month, 13% in the second month, and 7.4% in the third month. Specific symptoms and signs include pleural effusion (92.6%), pericardial effusion (88.9%), elevated inflammatory markers (74.1%), pleuritic chest pain (55.6%), fever (53.7%), and pericardial friction rub (32.3%) (14). The clinical manifestations of PCIS in children are similar to those in adults, with many cases presenting with fever as the initial symptom, often accompanied by loss of appetite and fatigue. However, because children's immune systems are more active, the symptoms can be more severe than in adults once the condition occurs (15).

## Diagnostic method

Early diagnosis is crucial for the management of PCIS) as it allows for timely intervention to prevent disease progression and reduce the risk of complications. The diagnostic criteria for PCIS include five features, of which at least two must be present: fever with no obvious alternative cause, pleuritic or pericarditis chest pain, pleural or pericardial friction rubs, pericardial effusion, and pleural effusion with elevated CRP (16). In most cases, the diagnosis of PCIS is made by exclusion. Table 1 shows diagnostic criteria for PCIS.

**Table 1:** Diagnostic criteria and treatment of post-cardiac injury syndrome.

Category	Details
Diagnostic criteria	Must meet at least two of the following features: (i) Fever without alternative cause (ii) Pleuritic or pericarditic chest pain (iii) Pleural or pericardial rub (iv) Evidence of pericardial effusion (v) Pleural effusion with raised CRP




First-line medications	Aspirin or NSAID + colchicine
Second-line medications	Corticosteroids
Third-line medications	Anakinra, Azathioprine, Intravenous immunoglobulins

NSAIDs, nonsteroidal anti-inflammatory drugs.

Preliminary tests include inflammatory markers such as Complete Blood Count (CBC), Erythrocyte Sedimentation Rate (ESR), C-reactive protein (CRP), and Troponin T or I. Although the sources of these markers are non-specific, their elevation in the absence of other diseases can suggest PCIS, as these markers are elevated in over 83% of PCIS patients (1).

Chest x-rays can help exclude causes of chest pain or respiratory difficulty such as pneumonia or pneumothorax, and in cases of PICS, some patients may show an enlarged cardiac silhouette. An electrocardiogram is the most important fundamental test for diagnosing active pericarditis in PCIS, with widespread ST segment elevation and PR segment depression across multiple leads warranting caution for post-cardiac injury syndrome. Transthoracic echocardiography (TTE) is a convenient and safe diagnostic tool used to assess cardiac anatomical structures, hemodynamics, and function (17). TTE can definitively determine the presence of pericardial effusion and provide accurate quantitative information for pericardiocentesis. However, TTE has limitations in observing pericardial anatomy. Multimodal imaging, including cardiac CT (CCT) and cardiovascular MRI (CMR), is widely used in visualizing complex cardiac structures and provides detailed information about cardiac anatomy, function, and metabolism (18).

Early diagnostic methods such as electrocardiography, echocardiography, cardiac magnetic resonance imaging, and biomarker testing each have their own advantages in the diagnosis of cardiac injury syndrome. By integrating the patient's clinical presentation and laboratory results, physicians can formulate personalized diagnostic strategies, thereby enhancing the accuracy and timeliness of diagnosis. This is crucial for early intervention and improving patient prognosis.

## Management

The management for PCIS aligns with the guidelines for diagnosing and managing pericardial diseases, with nonsteroidal anti-inflammatory drugs (NSAIDs) and colchicine as the first-line treatment options (19). Corticosteroids are alternatives for symptom control, while immunomodulators such as anakinra and azathioprine, and immunoglobulins are considered for further intervention. Because children's immune systems are more sensitive, medication dosage and selection are approached with greater caution. The use of corticosteroids in children is more cautious, as prolonged use may have negative effects on growth and development, and they are typically only considered when symptoms cannot be controlled by other means. For children with other immune-related diseases, more individualized immunosuppressive therapy may be required (20). NSAIDs are the preferred treatment for PCIS. Due to the need for secondary prevention in almost all patient's post-myocardial infarction, NSAIDs are widely chosen as the primary drugs. For patients requiring concurrent antiplatelet therapy, aspirin is the preferred choice regardless of the reason. Common treatment plans in PCIS involve aspirin dosages of 750–1,000 mg every 6–8 h, gradually reducing by 750–1,000 mg per week; ibuprofen dosages should be 600–800 mg every 6–8 h, reducing by 400–800 mg per week. NSAID treatment should continue for several weeks to months until symptoms subside or inflammatory markers return to normal.

Colchicine is recommended for other pericarditis conditions, such as acute pericarditis and recurrent pericarditis, and thus is also applicable for PCIS. For patients with stubborn or recurrent pericarditis, medium-dose corticosteroids [e.g., prednisone 0.25–0.50 mg/(kg·d)] may be considered, with subsequent gradual reduction. Studies have shown that the recurrence rate during follow-up for recurrent pericarditis treated with high-dose prednisone (1.0 mg/kg per day) is 64.7%, while the recurrence rate for low to moderate-dose prednisone (0.2–0.5 mg/kg per day) is 32.6%. This suggests that lower doses of corticosteroids may be safer and more effective in the treatment of recurrent pericarditis (21).

For steroid-dependent PCIS patients, immunomodulators (such as anakinra and azathioprine) and intravenous immunoglobulins are recommended, especially when colchicine treatment is ineffective (22, 23). The use of immunomodulatory treatment is not common among PCIS patients. For patients with recurrent pericarditis that does not respond to conventional treatment, stronger immunosuppressive therapies may be required to control the immune response (21).

Limiting strenuous physical activity is an important treatment measure, recommended until symptoms subside and inflammatory markers and imaging studies (ESR, CRP, ECG, TTE) return to normal. There are more standardized recommendations for athletes, who should restrict strenuous physical activities for at least three to six months (16). Chronic pericarditis is a relatively rare complication of PCIS. Although its incidence is not high, some PCIS patients may experience prolonged chronic inflammation of the pericardium. Additionally, as a non-pharmacological treatment option, pericardiectomy may be considered when drug treatment is ineffective (24).

## Prevention strategy and prognosis

Most preventative studies currently focus on PPS, identifying risk factors for PCIS such as younger age, type of surgery, history of corticosteroid use, past pericarditis, and lower BMI (25). Research has shown that colchicine significantly reduces the incidence of PCIS following cardiac surgery, while corticosteroids have not been proven to have a preventative effect (26, 27). Although the prognosis for most patients with post-cardiac injury syndrome is relatively good, studies report a recurrence rate of 10% to 15%. Given that about 0. 5% of PPS patients develop constrictive pericarditis, longer follow-up for PCIS patients is recommended (28).

## Conclusion

The main manifestation of PCIS is pericarditis. The disease is considered to be autoimmune-edited, with some individuals being more susceptible.

The key to treatment is anti-inflammatory interventions, with nonsteroidal anti-inflammatory drugs (NSAIDs), colchicine, and restricted physical activity as the preferred measures.

Although our understanding of this syndrome continues to grow, ongoing scientific exploration is needed to refine diagnostic criteria, optimize treatment plans, and further elucidate its mechanisms.
